# Effects of Hot-Air Drying Conditions on Quality Attributes of Meat and Shell of Dried Shrimp

**DOI:** 10.3390/foods14234041

**Published:** 2025-11-25

**Authors:** Zhongjing Lin, Zhaorong Zhang, Zhipeng Zheng, Ruoting Hou, Yi Zhang, Baodong Zheng, Natthida Sriboonvorakul, Jiamiao Hu

**Affiliations:** 1 College of Food Science, Fujian Agriculture and Forestry University, Fuzhou 350002, China; 2 Engineering Research Centre of Fujian-Taiwan Special Marine Food Processing and Nutrition, Ministry of Education, Fujian Agriculture and Forestry University, Fuzhou 350002, China; 3 Department of Clinical Tropical Medicine, Faculty of Tropical Medicine, Mahidol University, Bangkok 10400, Thailand

**Keywords:** *Litopenaeus vannamei*, hot-air drying, texture profile analysis, sensory evaluation, LM-ANN

## Abstract

Maintaining desirable texture, color, and flavor during hot-air drying is crucial for improving the commercial value of dried shrimp. This study aims to address the limitations of previous research on hot-air drying of shrimp, which focused solely on the meat. The objective is to simultaneously investigate the dual effects of hot-air drying conditions on the textural and physicochemical properties of both the shrimp shell and meat. This provides a theoretical foundation for preserving the optimal texture, color, and flavor of dried shrimp snack products. After drying and separation, the textural and physicochemical properties of the two components were comprehensively evaluated, including hardness, crispness, chewiness, springiness, color (L*, a*, b*), rehydration rate, sensory attributes, and odor characteristics. Furthermore, to elucidate the complex interrelationships among these variables, two predictive models were established: a Partial Least Squares Regression (PLSR) model and an Artificial Neural Network (ANN) model optimized using the Levenberg–Marquardt algorithm. The PLSR model achieved a calibration accuracy of R^2^ = 0.38 and a validation accuracy of R^2^ = 0.32, whereas the optimized LM-ANN model exhibited markedly superior predictive capability (R^2^_Training_ = 0.99, R^2^_Validation_ = 0.98), effectively capturing nonlinear associations between drying parameters and quality attributes of both meat and shell. Finally, a user-oriented prediction module was established based on the optimized ANN model, allowing flexible input of variables and prediction of quality outcomes. This integrated framework may provide a novel approach for modeling and optimizing the hot-air drying process of shrimp, offering practical guidance for quality control and texture customization of dried shrimp products.

## 1. Introduction

*Litopenaeus vannamei,* commonly known as the Pacific white shrimp or the South American white shrimp, typically has a translucent or light grey body with no distinct stripes or spots. Compared to other shrimp species, it demonstrates remarkable adaptability and rapid growth in marine environments [1]. Renowned for its sweet flavor and high meat-to-shell ratio, it is one of the world’s most valuable commercially exploited aquatic species [2]. Aquaculture production of *L. vannamei* accounts for 70 per cent of the total global aquaculture production [3]. In 2020, the global aquaculture production of *L. vannamei* reached approximately 5,812,180 metric tons [4]. Similar to other marine shrimp species, *L. vannamei* possesses a high moisture content and is prone to rapid spoilage due to both endogenous enzymatic activity and exogenous microbial contamination, thereby limiting its distribution in fresh form [5]. Consequently, freshly harvested shrimp should be promptly processed to extend shelf life. Drying is a traditional preservation technique. It remains one of the most effective methods for extending the shelf life of shrimp. This process not only minimizes microbial spoilage through moisture reduction but also helps retain the characteristic flavor and allows for ambient storage conditions [6]. Given its high yield, favorable growth characteristics, and consumer acceptance, *L. vannamei* has also become a major raw material for various processed shrimp products, including dried shrimp [7]. Dried shrimp represents one of the most common processed forms of shrimp, widely appreciated across global cuisines for its intense umami flavor and extended shelf life. The Asia-Pacific region, particularly countries such as South Asia and Southeast Asia, is the primary consumer market. This is due to a strong cultural preference for dried seafood [8,9]. Dried shrimp is a rich source of high-quality protein and essential minerals such as calcium, phosphorus, and potassium. It is widely consumed both as a ready-to-eat product and as an ingredient in culinary applications due to its low moisture content and desirable sensory attributes, including distinctive aroma, savory taste, and natural color. It can be used after rehydration or directly incorporated into soups, stir-fried dishes, and sauces, providing both nutritional value and enhanced flavor profiles in various traditional recipes [10].

Traditional dried shrimp production involves washing the fresh shrimp thoroughly, draining them, and sun drying them for a period in natural conditions before packaging them for storage or distribution. This process reduces the moisture content sufficiently to slow down the enzymatic reactions and other biological processes that cause spoilage [11]. Moreover, the drying process enhances the natural flavor of the shrimp while improving its texture and mouthfeel. Although sun drying has been used for centuries to produce dried shrimp, this method is still very weather-dependent. Adverse weather conditions such as rainfall, high humidity, and temperature fluctuations can hinder the drying process and compromise the quality of the final product [12]. Furthermore, sun drying outdoors exposes products to potential contaminants such as insects, microbial contamination, and heavy metals, posing significant risks to the sanitary safety of dried seafoods [13]. Zhu et al. compared the effects of five drying methods, including sun drying and hot-air drying, on the quality of processed fish fillets. The results showed that hot-air drying was significantly more effective than sun drying at improving the texture and flavor of processed aquatic products, while also enhancing processing efficiency [14]. Pongsetkul et al. investigated the feasibility of employing hot-air drying at varying temperatures as an alternative to traditional sun drying for producing salted shrimp paste. Compared to conventional sun drying, hot-air drying reduces enzyme activity and hydrolysable during fermentation while slowing the rate of browning [15]. This clearly demonstrates the superiority of controlled drying processes in preserving color quality. Meanwhile, current comparative studies predominantly focus on isolated visual attributes or metrics, paying insufficient attention to variations across different tissue types within dried shrimp. This hinders the precise optimization and advancement of drying processes. Consequently, there has been a growing interest in controlled drying techniques designed to overcome these limitations and produce dried shrimp with enhanced quality and reliability. Among various dehydration techniques, hot-air drying is extensively utilized in the food industry due to its ability to provide controlled environmental conditions. This method employs heated air as the drying medium, facilitating moisture removal from both the surface and interior of the product through convective circulation [16]. Consequently, hot-air drying effectively preserves quality attributes and has demonstrated considerable efficacy in enhancing the quality of dried shrimp products [17]. For instance, Xu et al., determined the drying kinetics of shrimp using this method and proposed a real-time monitoring approach for assessing quality changes in dried shrimp, based on key quality parameters observed during the hot-air drying process [18]. Moreover, Indriani et al., investigated the moisture loss during the hot-air drying process and found that drying treatment induces protein oxidation and degradation in shrimp, leading to a gradual breakdown into small peptides [19]. These changes play a critical role in determining the sensory quality of dried shrimp.

While these studies demonstrate the effectiveness of modern dehydration techniques in producing dried shrimp, some studies primarily concentrate on the impact of drying parameters on the meat of the shrimp itself [10]. This suggests that the differences between the various tissues of dried shrimp have been overlooked. Notably, dried shrimp are usually eaten whole, including the shell, and do not require further processing. Shell-on dried shrimp samples have been widely used in research into drying processes, demonstrating that they represent a crucial research category [20,21,22]. Shrimp shell significantly impacts product texture, with crispness being a key quality attribute, while color notably influences consumer appeal, among other factors. As far as we know, no systematic research has yet examined the dual effects of drying conditions on the texture of both the shell and the meat of dried shrimp. Furthermore, mathematical modelling approaches to analyze the differentiation of dried shrimp tissue quality attributes from a multivariate perspective are lacking. This makes it difficult for us to refine the process parameters in dried shrimp production with greater precision.

Therefore, this study focuses on *Litopenaeus vannamei* as the subject of the research. It specifically examines the quality differences between the shrimp shell and meat obtained after processing whole shrimp under identical hot-air drying conditions, including moisture content, textural properties, color difference, rehydration rate, electronic nose analysis, and sensory evaluation. Mathematical modelling techniques were employed, with partial least squares regression (PLSR) and Levenberg–Marquardt algorithm optimization of an artificial neural network (LM-ANN) used for comparative analysis. PLSR can effectively identify collinearity issues among independent variables and investigate the linear relationships between drying indicators and parameters during the drying process of dried shrimp. Traditional analytical methods struggle to accurately capture the intricate interplay of multiple factors when deciphering the response mechanisms of shrimp shell and meat tissues to hot-air drying temperatures. The LM-ANN has enhanced non-linear fitting capabilities, and the LM-ANN overcomes the limitations of conventional linear models to delve deeply into the latent correlations within the data [23]. LM-ANNs applied within the food industry employ machine learning models characterized by high predictive capability and accuracy, representing a significant developmental direction within the field of food science [24]. Consequently, it was selected as the core analytical tool for this study and was used to quantitatively analyze the tissue-specific responses at different temperatures. The structural characteristics of shrimp shell and meat exhibit fundamental differences: the former is densely compacted while the latter is comparatively loose. Their primary constituents are chitin and proteins with water, respectively. It is hypothesized that their physicochemical properties will change differently under varying drying temperatures. It is anticipated that the shrimp carapace will exhibit a more gradual response to temperature variations, whereas the muscle tissue may undergo more pronounced protein degradation and lipid oxidation reactions at higher temperatures. The LM-ANN is expected to effectively capture this tissue-specific temperature response mechanism, thereby enabling precise prediction of dried shrimp quality indicators. The findings of this study provide an intuitive illustration of how variations in drying conditions affect the quality of dried shrimp. Synthesizing these approaches enables the system to comprehensively elucidate the quality transformation patterns of shrimp shell and meat during hot-air drying. This provides a theoretical foundation and a reliable tool for optimizing production processes and enhancing the quality control of dried shrimp snacks.

## 2. Materials and Methods

### 2.1. Materials

The shrimp (*Litopenaeus vannamei*) used in the experiment were procured from a local seafood market (Fuzhou, China), with an average length of 7–8 cm and a weight range of 11–13 g per shrimp. The samples were carefully chosen based on their vibrant coloration, intact limbs, normal odor, and resilient texture. The pH value of muscle tissue was measured using a pH meter (Mettler-Toledo Instruments (Shanghai) Co., Ltd., Shanghai, China, FE20) to verify freshness. This yielded a result of 7.64 ± 1.51. This value falls within the typical pH range for fresh white prawns [25]. Subsequently, samples that passed verification were washed to remove impurities from the prawn’s surface. The samples were stored in a laboratory fridge at 4 °C for subsequent use. The initial moisture content of the shrimps was 75.18 ± 1.40% (wet basis, w.b.) as an average of the results obtained, consistent with values reported in comparable studies [26]. Before the drying process, the fresh shrimp were thoroughly washed, drained, and beheaded to ensure the uniformity of the samples, improve the drying efficiency, and extend the shelf life. Previous studies using hot-air drying have used a similar pretreatment procedure [5,10]. The drying experiment commenced with whole shrimp samples, recording data in real time to plot drying curves and calculate drying rates. Following the conclusion of the drying process, it is imperative that the dried shrimp is permitted to attain room temperature in the laminar flow hood prior to commencing the manual separation process. Initially, the scissors must be disinfected by wiping them with 75% alcohol. Operators wore sterile gloves throughout the procedure. First, drying curves and drying rates were determined for whole shrimp with unseparated shell and meat. Subsequently, shell and meat were manually separated. Under each condition, both shrimp shell and shrimp meat were sampled individually (unmixed) in 25 replicates each. Five replicates were shared for visual inspection and color difference analysis (non-destructive), with the remaining samples used for rehydration ratio, sensory evaluation, e-nose analysis, and TPA (destructive); each utilized five replicates. Color difference analysis, TPA, rehydration ratio, sensory evaluation, and e-nose analysis were then conducted separately on the shell and meat. All experimental data were utilized for subsequent mathematical model construction (Table 1). All other chemicals and reagents were of analytical grade.

### 2.2. Hot-Air Drying (HAD)

After pre-treatment, the shrimp were arranged on drying trays measuring 400 mm × 300 mm and placed in a drying oven equipped with a forced-air ventilation system (DHG, Shanghai Jinghong Experimental Equipment Co., Ltd., Shanghai, China) for drying experiments. During the hot-air drying process, the shrimp were turned every 20 min to ensure even heating on both sides. Drying temperatures were set at 60, 70, 80, 90, and 100 °C until the moisture content of the samples decreased to approximately 15–20% (w.b.). The permissible deviation for each set temperature is ±1 °C. During the experiment, measurements were taken using a thermo-hygrometer (HTC-1, humidity ± 5% RH) positioned near the drying oven’s air inlet. The relative humidity range within the laboratory environment was 48.2% to 55.6% RH. During drying, the relative humidity within the chamber decreased progressively with increasing temperature, ranging overall from 11.3% to 26.2%% RH. The airflow velocity at the sample placement level was set to 1.0 m/s, a parameter referenced from research on hot-air drying of shrimp [7]. Verification through shrimp drying experiments confirmed that this air velocity ensures uniform and stable airflow within the chamber. This approach balances drying efficiency for dried shrimp while minimizing quality issues such as surface shrinkage and nutrient loss, thereby complying with application standards for forced convection forced-air drying ovens in food drying.

### 2.3. Moisture Content Determination

The moisture content refers to the weight of the water in a product, and it can be presented on a wet matter basis. The moisture content was determined by the direct drying method. The shrimp samples were placed in a halogen moisture analyzer (DSH-50A, Shanghai Yoke Instrument Co., Ltd., Shanghai, China) and dried at a temperature of 105 °C. The measurement was recorded at the end of the drying process after the removal of surface moisture. Equation (1) was then used to determine the shrimp’s moisture content. When the desired moisture content (20% or 15% (w.b.)) was reached, the drying process was stopped.

(1) $$M_{w}\left(\%,\;w.b.\right)=\frac{m_{0}-m_{1}}{m_{0}}\times 100\%$$

where $M_{w}$ (%) represents the moisture content in percentage, $m_{0}$ (g) denotes the initial mass of the material, and $m_{1}$ (g) signifies the mass of the solid matrix after drying at 105 °C.

### 2.4. Drying Rate (DR)

The drying rate (*DR*) of the sample was calculated using Equation (2) based on the moisture content of the shrimp at different time points.

(2) $$\;\mathrm{DR}=\frac{M_{t}-M_{t+\Delta t}}{\Delta t}$$

where $M_{t}$ is the wet basis moisture content of the sample at time point *t* (g/g), $M_{t+\Delta t}$ represents the wet basis moisture content of the shrimp at time point t+Δt (g/g), and Δt is the time interval (in minutes) between two consecutive measurements of the wet basis moisture content of the shrimp. The mass loss during the drying process was recorded at 20 min intervals.

### 2.5. Color Measurements

In color measurement, the color of shrimp meat is assessed based on the second and third abdominal segments, where meat coloration is relatively uniform and less susceptible to pigment variations than in the head and tail regions. The pigmentation of the exoskeleton is evaluated separately using shrimp analysis. The color parameters of dried shrimp were determined using a colorimeter (WSC-3B, Shanghai INESA Physico-Optical Instrument Co., Ltd., Shanghai, China) and calibrated with a white standard tile. The chromaticity parameters of the white standard plate used are L* = 94.0 ± 0.5, a* = 0.9 ± 0.4, b* = 0.5 ± 0.2. The Hunter Lab color system quantifies color through three-dimensional parameters: L* (0 denotes black, 100 denotes white), a* (chromatic opposition between red (+a) and green (−a)), and b* (chromatic opposition between yellow (+b) and blue (−b)) [27].

### 2.6. Texture Profile Analysis (TPA)

The textural properties of dried shrimp samples were determined using a texture analyzer (Universal TA, Shanghai Tengba Instrument Technology Co., Ltd., Shanghai, China). Probes with a cylindrical shape were used, including P/50 (50 mm diameter) and a P/5 (5 mm diameter) [28]. The analysis was performed using texture profile analysis (TPA), and the operating conditions were as follows: compression degree was 30%, with probe pre-test speed, test speed, and post-test speed set to 1.0 mm/s. Textural parameters are calculated by the software, including the determination of dry shrimp hardness, crispness, springiness, and chewiness.

### 2.7. Rehydration Ratio

A measured quantity of shrimp shell and shrimp meat samples was taken, based on existing rehydration studies of dried foods with slight modifications [29,30], and placed in an 80 °C constant-temperature water bath for 15 min. Before weighing, any residual surface moisture was removed using filter paper. The ability of the dried shrimp to absorb water and return to its fresh state is characterized by the rehydration ratio, which is the ratio of the weight at that time to the initial weight. Equation (3) was used to determine the rehydration ratio (RR). The weight of each shrimp sample of used in the experiment was 2 ± 0.05 g.

(3) $$\;\mathrm{RR}=\frac{m_{e}}{m_{0}}$$

where $m_{e}$ is the weight (g) of the shrimp samples after rehydration and $m_{0}$ is the weight of the shrimp samples before rehydration.

### 2.8. Sensory Evaluation

The Quantitative Descriptive Analysis (QDA) method was used to evaluate the sensory characteristics of the dried shrimp samples, with slight modifications to the method reported by Di et al. [31]. The sensory panel consisted of 5 trained members between the ages of 22 and 30, all from the College of Food Science, Fujian Agriculture and Forestry University (Fujian, China). All evaluators participated in the experiment on a voluntary basis, having signed informed consent forms, and adhered to relevant protocols throughout the process. No personal privacy information was collected, and participants’ fundamental rights and privacy were protected. Before the assessment, the panel was systematically trained to standardize their understanding of the sensory descriptors specific to dried shrimp, as shown in Table S1. The total training duration was 8 h, ensuring all assessors maintained consistent criteria for judging sensory indicators. The sensory evaluation focused on six primary aroma attributes: metallic, sweet, cooked-meat-like, fishy, caramel, and roasted/nutty. These attributes were used to quantitatively describe the characteristics of both shrimp shell and meat. A rest period of 5 min was provided between evaluations to minimize sensory fatigue. The numerical scale employed is a 0 to 5 system, with 0 indicating no perception and 5 indicating a very strong perception, using increments of 0.1.

### 2.9. E-Nose Analysis

The experimental methodology of the electronic nose was based on the literature of Sun et al. with slight modifications [32]. The flavors of different parts of dried shrimp were evaluated using an electronic nose (PEN 3, Airsense Analytics, Schwerin, Germany). The PEN3 system’s electronic nose is equipped with ten metal oxide sensors (MOS), which respond selectively to the characteristic volatiles of different types of fragrance (Table S2). Prior to e-nose detection, baseline calibration and drift correction were performed. Ambient air filtered through activated carbon served as the blank baseline; after introducing it into the sensor chamber and stabilizing for 60 s, the baseline signal was recorded. A sliding window method was employed to subtract drift components in real time. After completing the above calibration, samples were equilibrated for 30 min at room temperature within sealed bottles to ensure the release of volatile components. During testing, sensor response signals were continuously sampled for 60 s to obtain relative change values for each sensor. Response values (G/G_0_) from all three sensors were collected during the curve stabilization phase, with the average serving as the result.

### 2.10. Microbiological Testing

After accurately weighing 10.0 g of dried shrimp sample, 90 mL of sterile physiological saline (0.85% NaCl) was added to prepare a 1:10 dilution. Then, 0.1 mL of the appropriate dilution was taken and spread onto Plate Count Agar (PCA). This was incubated at 37 °C for 48 h and the total colony-forming units (CFU/g) were counted. An equal volume of the dilution was taken and mixed thoroughly with Crystal Violet-Neutral Red-Bile Salt Agar (VRBA), then incubated at 37 °C for 24 h, and the typical colonies of coliform bacteria were counted. Both PCA and VRBA media were procured from Guangdong Huankai Microbial Sci. & Tech Co., Ltd. (Guangzhou, China).

### 2.11. Correlation Analysis

Pearson correlation analysis was employed to examine the linear relationships among various quality indicators of dried shrimp under different drying conditions. The variables analyzed included drying temperature, moisture content, rehydration ratio, color difference indices, and textural properties. Correlation coefficients r ranged between −1 and 1, with higher absolute values indicating stronger correlations. The significance level was set at *p* < 0.05. Correlation heatmaps were generated to visualize the strength of correlations. Pearson correlation analysis is a method of preliminary exploration that seeks to identify the associative characteristics between indicators from a linear perspective. Subsequent LM-ANN modelling then focuses on capturing non-linear features among variables, forming a complementary analysis.

### 2.12. Partial Least Squares Regression

Tissue type (*X*_1_), temperature (*X*_2_), and moisture content (*X*_3_) were used as independent variables in the raw data matrix, while texture characteristics (hardness, crispness, springiness, and chewiness), color parameters (L*, a*, b*), rehydration ratio, sensory evaluation, and electronic nose results were specified as response variables (*Y*) as input objects. The general form of the PLSR model is shown in Equation (4). The specific values are presented in Tables S3 and S4. PLSR can simultaneously process datasets with multiple inputs and outputs. The modelling dataset was divided into a calibration set, which was used for model training, and a validation set, which was used to determine the number of latent variables through k-fold cross-validation.

(4) $$Y_{i}=b_{0}+b_{1}\cdot X_{1}+b_{2}\cdot X_{2}+b_{3}\cdot X_{3}$$

where *b* denotes the regression coefficients, comprising the intercept and the regression coefficients of the independent variables.

### 2.13. Levenberg–Marquardt Algorithm Optimized Artificial Neural Network (LM-ANN)

**Figure 1:**
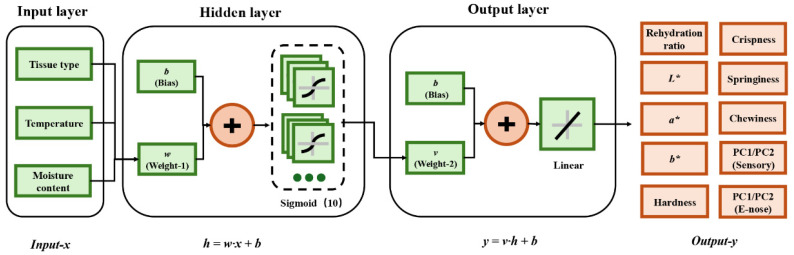
Schematic diagram of the LM-ANN architecture.

The artificial neural network structure consists of an input layer, a hidden layer, and an output layer, the input layer contains three independent variables including tissue type, drying temperature (°C), and drying time (min), the hidden layer is set to contain 10 neurons with a Sigmoid activation function to introduce nonlinear feature mapping, and the output layer includes 12 response variables that including hardness, crispness, chewiness, springiness, color difference (L*, a*, b*), and rehydration ratio. The output layer uses a linear activation function to achieve continuous value prediction (Figure 1). The dataset was divided into training, validation, and test sets with 70%, 15% and 15% set parameters, respectively. The neural network was trained iteratively by Levenberg–Marquardt algorithm (Table 2). Prior to model training, all input and output variables underwent min-max normalization to eliminate differences in magnitude between variables. The random seed was fixed at 1 for all experiments to ensure the reproducibility of the artificial neural network (ANN) model training. The LM-ANN model employs a multi-input, multi-output architecture, enabling the simultaneous incorporation of multiple process parameters as input variables and the generation of prediction outputs for several quality indicators. It transmits signals through neural layers, undergoes weighted summation and processing via non-linear activation functions, ultimately producing multi-indicator output signals to achieve precise mapping. The output formula for a single hidden layer neuron and output layer neuron in the LM-ANN is shown in Equation (5). The LM-ANN model was constructed using the Neural Network Toolbox in MATLAB (R2023a). Additional code was implemented to enable variable input and predict the quality of dried shrimp (Doc. S1).

(5) $$h_{j}=f\;\left(\sum_{n=1}^{p}w_{\mathrm{ji}}\cdot x_{i}+b_{j}\right),y_{k}=g\;\left(\sum_{n=1}^{q}v_{\mathrm{kj}\;}{\cdot h}_{j}+b_{k}\right)$$

where *x_i_* denotes the input variable, *h_j_* denotes the output value of the *j*-th hidden layer neuron, *y_k_* denotes the output value of the *k*-th output layer neuron, *w_ji_* is the weight value from the *i*-input layer variable to the *j*-th hidden layer neuron, *v_kj_* denotes the weight from the *j*-th variable in the hidden layer to the *k*-th neuron in the output layer, *b_j_* and *b_k_* are the bias values adjusting the activation thresholds of neurons in different layers, f(x) is the nonlinear activation function, g(x) is the linear activation function, and n is the number of neurons.

### 2.14. Statistical Analysis

The data analysis was processed using SPSS software (version 27.0, SPSS Inc., Armonk, NY, USA). The mean values were performed using one-way ANOVA followed by Duncan’s multiple comparisons test. *p* < 0.05 was considered a statistically significant difference. The experimental data were processed using GraphPad Prism 10.0 (GraphPad Software, Boston, MA, USA) and Origin 2024b (OriginLab Corporation, Northampton, MA, USA) software for figure plotting. Principal component analysis (PCA) was performed on sensory evaluation and electronic nose data using Origin 2024b to reduce dimensionality and simplify the dataset through multidimensional statistical processing. PLSR modelling of dried shrimp data was performed using The Unscrambler X software (Version 10.4, CAMO Software AS, Oslo, Norway). Levenberg–Marquardt algorithm-optimized ANN modelling was performed using MATLAB R2023a Neural Network Toolbox (MathWorks, Natick, MA, USA) to capture the potential non-linear relationship between drying conditions and shrimp characteristics. Furthermore, to investigate the relationships among multiple variables in dried shrimp, the drying temperature, the moisture content and the tissue type of dried shrimp tissue (encoded as numerical variables for the model calculations, where 0 denotes the shell and 1 denotes the meat) were set as the independent variables. The response variables for subsequent mathematical modelling and discriminant analysis were color difference (L*, a*, b*), textural properties (hardness, springiness, chewiness, and crispness), rehydration ratio, the first two principal components (PC1 and PC2) of sensory characteristics, and electronic nose characteristics. The coefficient of determination (R^2^) and root mean square error (RMSE) were adopted as evaluation metrics for the predictive performance of the LM-ANN and PLSR models, as expressed in Equations (6) and (7) below. To ensure consistency in comparing PLSR and LM-ANN models, both PLSR’s R^2^_Calibration_ and LM-ANN’s R^2^_Training_ are used to evaluate model fitting capability on training data. Both PLSR’s R^2^_Validation_ and LM-ANN’s R^2^_Validation_ are employed to assess model stability and resistance to overfitting during parameter tuning. All measurements were taken in five independent replicates. Each repetition uses one shrimp or its tissue as an independent sample unit, and the results were reported as the mean ± standard deviation (Mean ± SD).

(6) $$R^{2}=1-\frac{\sum_{i=1}^{n}{\left(y_{i}-{\hat{y}}_{i}\right)}^{2}}{\sum_{i=1}^{n}{\left(y_{i}-{\bar{y}}_{i}\right)}^{2}}$$

(7) $$RMSE=\sqrt{\frac{\sum_{i=1}^{n}{\left(y_{i}-{\hat{y}}_{i}\right)}^{2}}{n}}$$

where *i* denotes the sample index, n denotes the number of samples, $y_{i}$ denotes the actual value, ${\hat{y}}_{i}$ denotes the predicted value, and ${\bar{y}}_{i}$ denotes the mean of the actual values. R^2^ ranges from 0 to 1, with values closer to 1 indicating better model fit. RSME ranges above 0, and smaller values signify higher prediction accuracy.

## 3. Results

### 3.1. Characteristics of the Hot-Air Dring Curve for Whole Shrimp

**Figure 2:**
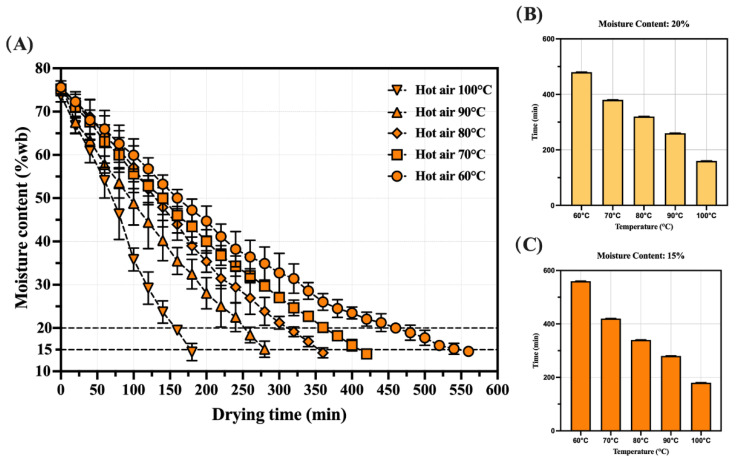
Drying curves of dried shrimp at different hot-air temperatures. (**A**) The variation of shrimp moisture content as a function of time. (**B**) Comparison of drying time for samples at different hot-air temperatures (moisture content of 20%). (**C**) Comparison of drying time for samples at different hot-air temperatures (moisture content of 15%).

The changes in the moisture content and drying rate of shrimp during the hot-air drying process are shown in Figure 2. Initially, the moisture content of fresh shrimp is 75.18 ± 1.40%. The moisture content primarily exhibits a decreasing trend with the extension of drying time (Figure 2A). As the drying temperature increased (60, 70, 80, 90, and 100 °C), the time required to reach a moisture content of 20% to 160, 260, 320, 380, and 480 min, respectively. When targeting a lower final moisture content of 15%, the drying times extended to 180, 280, 340, 420, and 560 min, respectively (Figure 2B,C). The amount of free water on the shrimp’s surface is comparatively high during the first stages of drying, and it gradually evaporates as drying goes on; a greater temperature accelerates this process. The drying rate curve exhibits a trend similar to that reported in previous studies [26].

### 3.2. Drying Rate Characteristics of Whole Dried Shrimp for Hot-Air Drying Treatment

**Figure 3:**
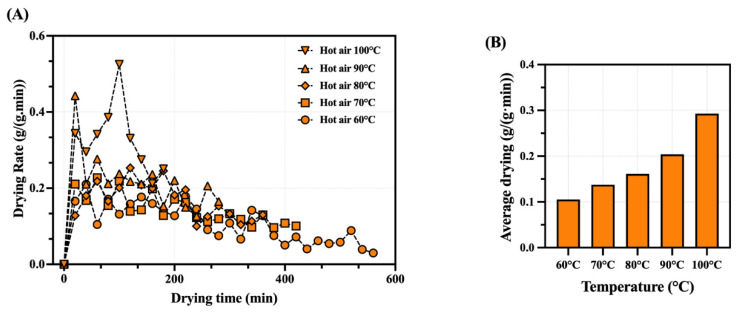
Drying rate of whole dried shrimp under different drying conditions. (**A**) The variation of shrimp average drying rate as a function of time. (**B**) Comparison of drying rates for samples at different hot-air temperatures.

In the early stage, abundant surface free water and rapid heat transfer caused drying rate fluctuations. As moisture decreased, the removal of bound water slowed the rate progressively (Figure 3A). Although the overall drying rate exhibited a general trend of increasing initially and decreasing later, fluctuations were observed throughout the drying process. These irregular variations may be attributed to the complex water distribution in shrimp tissues. The variation in peak drying rates at different temperatures may be attributed to the temperature’s impact on the rate of water evaporation, with the average drying rates depicted in Figure 3B. In the later stages of drying, the moisture content decreases more slowly due to the tight binding of bound water with the tissue, which is not easily removed by drying, leading to a weakened dewatering capability. Xu et al. have demonstrated a similar trend in the results of hot-air drying for shrimp [18].

### 3.3. Morphological Observation of Whole Dried Shrimp and Its Separate Shell/Meat

**Figure 4:**
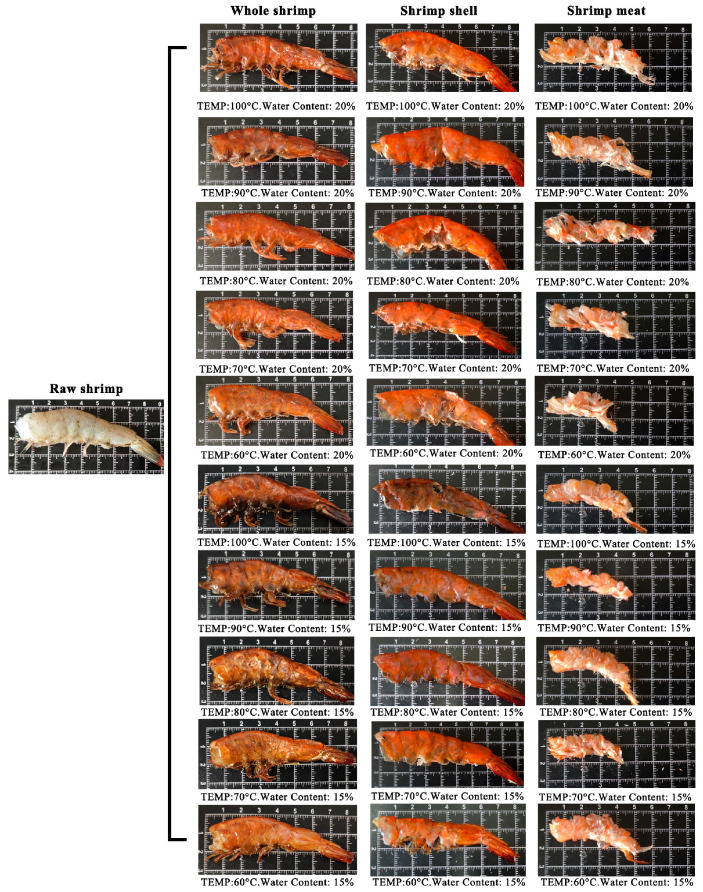
The images of whole dried shrimp and its separate shell/meat before and after treatment under different hot-air drying conditions.

The visual changes in whole shrimp, including the shell and meat, under varying drying conditions are shown in Figure 4. As the temperature increases, the color of the whole shrimp shifts gradually from light grey to red and ultimately to dark brown. The shell darkens while retaining its structure and developing a brittle texture. However, the color of the shrimp meat shows no significant variation, exhibiting a tendency towards volume reduction and increased hardness. If the temperature is set excessively high, scorching may occur on the surface of the shrimp.

### 3.4. Color Difference Analysis of Dried Shrimp Shell and Meat

**Figure 5:**
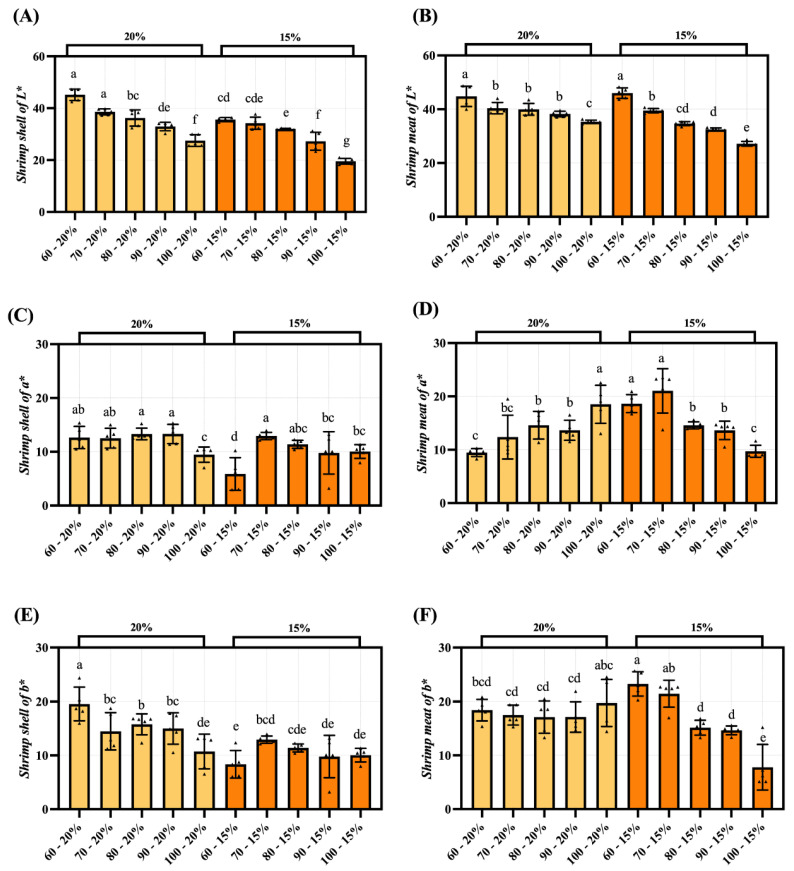
The impact of hot-air drying on the color of dried shrimp shell and meat. (**A**) Shrimp shell of L*. (**B**) Shrimp shell of a*. (**C**) Shrimp shell of b*. (**D**) Shrimp meat of L*. (**E**) Shrimp meat of a*. (**F**) Shrimp meat of b*. Groups not sharing a letter differ significantly at *p* < 0.05 (one-way ANOVA, Duncan’s test). The triangular markers in the bar chart denote measured values.

The results regarding the impact of drying temperature and moisture content on the color parameters of dried shrimp are shown in Figure 5. As the hot-air drying temperature increases and the duration extends, the dried shrimp surface darkens and loses brightness because of the Maillard reaction, which lowers the L* value (lightness). The characteristic browning in dried shrimp primarily arises from Maillard reactions between endogenous reducing sugars and amino acids during dehydration [10]. The a* and b* values of shrimp shell show a decreasing trend (Figure 5A,C,E). This may be due to the decrease in astaxanthin and carotenoid content in the shell caused by heat treatment. Shrimp meat may have unstable color changes due to its complex internal components, such as lipid oxidation and enzymatic reactions (Figure 5B,D,F). Under certain drying conditions, the a* and b* values of the dried shrimp meat increase, likely due to the decomposition of carotenoids during heating, leading to the release of astaxanthin [10]. Astaxanthin in shrimp shell can be released during heat treatment and bind to proteins in the shrimp meat. This causes a change in the color of the shell and meat.

### 3.5. TPA of Dried Shrimp Shell and Meat

**Figure 6:**
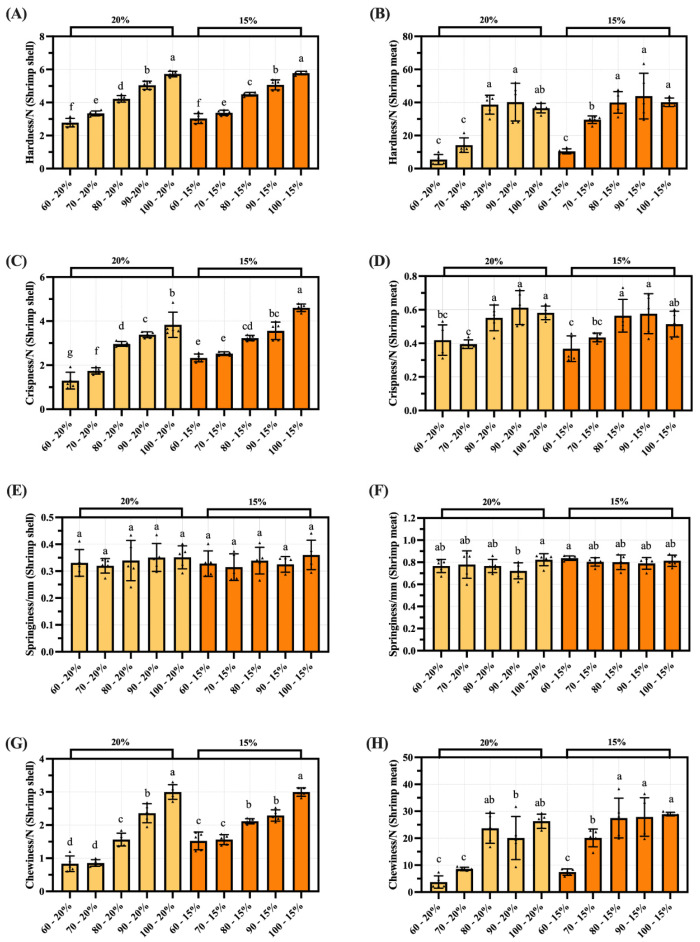
The effect of hot-air drying on the textural properties of dried shrimp shell and meat. (**A**) Hardness of the shrimp shell. (**B**) Hardness of the shrimp meat. (**C**) Brittleness of the shrimp shell. (**D**) Brittleness of the shrimp meat. (**E**) Springiness of the shrimp shell. (**F**) Springiness of the shrimp meat. (**G**) Chewiness of the shrimp shell. (**H**) Chewiness of the shrimp meat. Groups not sharing a letter differ significantly at *p* < 0.05 (one-way ANOVA, Duncan’s test). The triangular markers in the bar chart denote measured values.

The textural changes of the shrimp samples during the drying process are shown in Figure 6. The hardness of the shrimp shell increased from 2.78 N to 5.78 N, while the hardness of the shrimp meat rose from 5.48 N to 40.11 N (Figure 6A,B). The brittleness of the shrimp shell increased proportionally with drying temperature, rising from 1.30 N to 4.61 N (Figure 6C). Although the brittleness of the shrimp meat was lower than that of the shell, it also exhibited an upward trend with drying temperature, increasing from 0.37 N to 0.61 N (Figure 6D). The elasticity values for both the shrimp and meat fluctuated within ranges of 0.31 N to 0.36 N and 0.72 N to 0.84 N respectively, exhibiting no pronounced overall trend (Figure 6E,F). Both the chewiness of the shrimp shell and meat continued to increase during drying, with values ranging from 0.83 N to 3.00 N and 3.74 N to 28.94 N respectively (Figure 6G,H). This indicates that hot-air drying primarily enhances the density and toughness of both the shell and meat. Compared to the shrimp shell, the meat exhibits higher overall structural strength, with this treatment having minimal impact on its elasticity.

### 3.6. The Impact of Hot-Air Drying Temperature on the Rehydration Ratio of Dried Shrimp Shell and Meat

**Figure 7:**
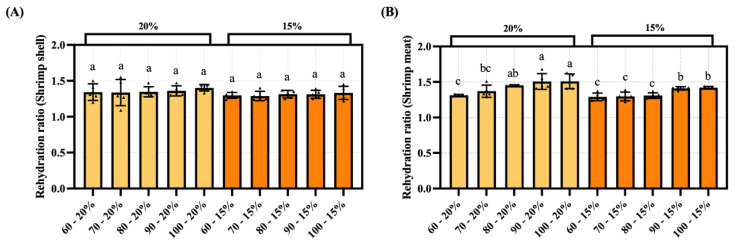
Rehydration rate of dried shrimp shell and meat under different hot-air drying conditions. (**A**) Shrimp shell. (**B**) Shrimp meat. Groups not sharing a letter differ significantly at *p* < 0.05 (one-way ANOVA, Duncan’s test). The triangular markers in the bar chart denote measured values.

As temperature increased and moisture content decreased, the rehydration rate of the shrimp shell showed no significant variation, ranging between 1.29 and 1.40 (Figure 7). Conversely, the rehydration rate of the shrimp meat exhibited a gradually increasing trend, peaking at 1.51. This suggests that shrimp meat has better rehydration properties than shrimp shell after hot-air drying. It is noteworthy that at the same temperature, the rehydration rate at 15% moisture content consistently falls below that at 20% moisture content, exhibiting an overall downward trend.

### 3.7. Sensory Evaluation of Dried Shrimp Shell and Meat

**Figure 8:**
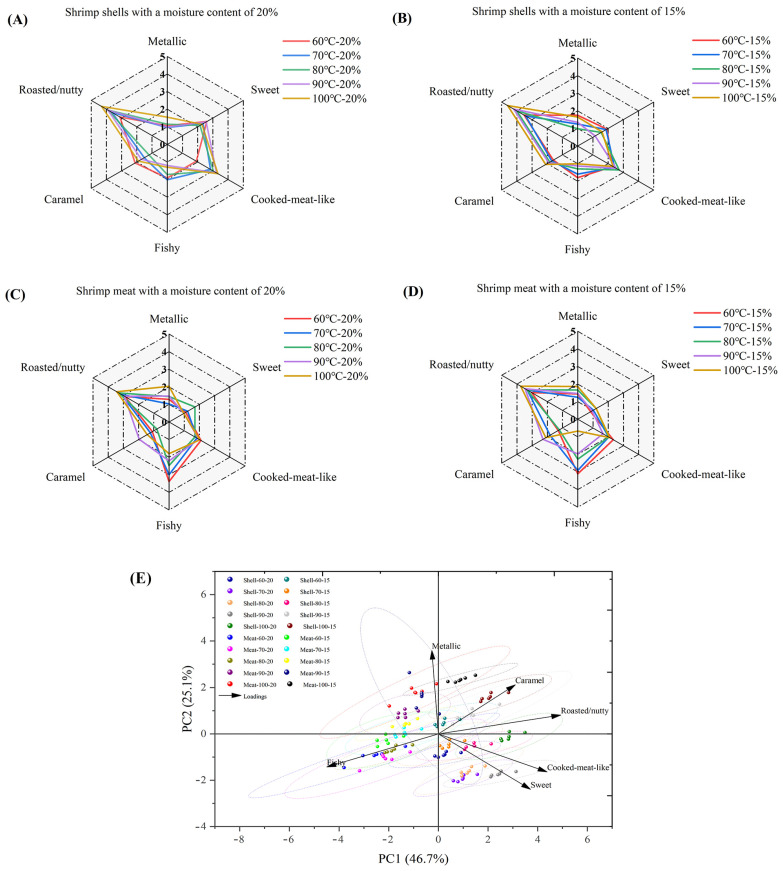
Sensory characteristics of hot-air-dried shrimp shell and meat. (**A**) Shrimp shell moisture content 20%. (**B**) Shrimp shell moisture content 15%. (**C**) Shrimp meat moisture content 20%. (**D**) Shrimp meat moisture content 15%. (**E**) PCA of sensory indicators of shrimp shell and shrimp meat.

Radar charts were used to compare the six core sensory attributes (metallic, sweet, cooked-meat-like, fishy, caramel, and roasted/nutty) of the four sample groups visually (Figure 8A–D). The results indicate that the aroma profiles of dried shrimp samples from identical tissues exhibited greater similarity at different drying temperatures. Notably, the roasted/nutty flavor profile of whole shrimp shell was more pronounced during hot-air drying, peaking at 4.6/5.00 at 100 °C with 15% moisture content. In contrast, shrimp meat released a certain quantity of aromatic compounds during processing (3.75/5.00), but exhibited accompanying fishy notes at lower temperatures, peaking at 3.39/5.00. This suggests that, while shrimp meat releases savory characteristics, it also carries a degree of fishiness. These discrepancies reflect flavor differences between distinct tissues within dried shrimp. After standardizing the sensory attribute data, principal component scores were calculated and the first two principal components (PC1 and PC2) were extracted, explaining 71.8% of the total variance (PC1: 46.7%, PC2: 25.1%) (Figure 8E). The results of the feature load analysis showed that the PC1 direction was primarily driven by positive indicators such as roasted/nutty and sweet (load values of 0.53 and 0.40, respectively), while the PC2 direction was strongly correlated with metallic (load value of 0.67). Dried shrimp samples are concentrated in the positive PC1 and negative PC2 regions (e.g., Shell-90-20, Shell-100-20), exhibiting stronger overall sensory advantages. This reflects how the overall aroma characteristics of dried shrimp change with temperature.

### 3.8. E-Nose Analysis of Dried Shrimp Shell and Meat

**Figure 9:**
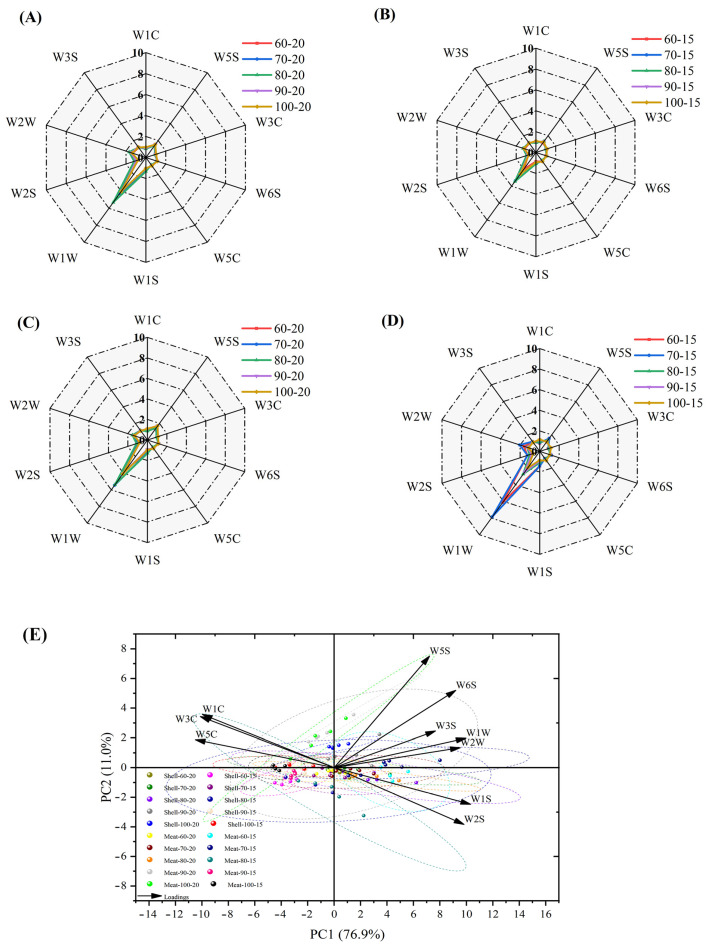
Electronic nose detection of the odor characteristics of hot-air-dried shrimp shell and meat. (**A**) Shrimp shell with 20% moisture content. (**B**) Shrimp shell with 15% moisture content. (**C**) Shrimp meat with 20% moisture content. (**D**) Shrimp meat with 15% moisture content. (**E**) Principal component analysis.

The response of each sensor during the hot-air drying process under different moisture content and temperature conditions is shown in Figure 9A–D. The results indicate that the W1W, W2W, and W5S sensors are highly sensitive to changes in the aroma of dried shrimp samples. The distribution patterns of shrimp shell and meat are similar within the overall odor profile, though this is more pronounced on the W1W sensing element, which primarily detects aromatic compounds. However, at moisture contents below 15%, shrimp meat exhibited a higher response intensity than the shell, peaking at 7.85/10.00 at 70 °C and 15% moisture content. As the temperature of the hot-air increased (from 90 to 100 °C), the response values of the W1W sensor gradually decreased. PCA was performed on the response data of the electronic nose sensor, and the cumulative contribution rate of the first two principal components, PC1 and PC2, reached 87.9% (PC1: 76.9%, PC2: 11.0%) (Figure 9E). PC1 is primarily contributed by sensor W5S, which is sensitive to amine compounds (load factor: 0.33), reflecting the distinct fishy odor present in different dried shrimp samples, which is associated with odor compounds produced during protein degradation; PC2 is associated with sensor W2W, which is highly responsive to alcohols and sulphury compounds (load factor: 0.50), substances typically produced during the spoilage of crustacean seafood. The Meat-90-15 and Shell-60-15 groups have negative projections on the W5S and W2W principal components, indicating relatively lower levels of amine- and sulphury-related off-flavors, resulting in a more advantageous overall flavor profile.

### 3.9. Analysis of Microbiological Indicator Test Results for Dried Shrimp Shell and Meat

**Figure 10:**
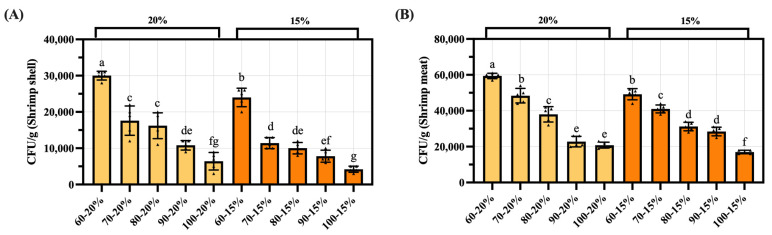
Total colony count results for shrimp shell and meat under different drying conditions. (**A**) Shrimp shell. (**B**) Shrimp meat. Groups not sharing a letter differ significantly at *p* < 0.05 (one-way ANOVA, Duncan’s test). The triangular markers in the bar chart denote measured values.

Microbiological testing results for dried shrimp samples indicate that the total plate-count bacteria in shrimp shell were 4.2 × 10^3^ ± 8.4 × 10^2^ to 3.0 × 10^4^ ± 1.2 × 10^3^ CFU/g, with the total plate-count bacteria in meat being 1.7 × 10^4^ ± 1.0 × 10^3^ to 5.94 × 10^4^ ± 1.52 × 10^3^ CFU/g (Figure 10). In the VRBA test for typical coliform colonies, shrimp shells or shrimp meat yielded undetectable levels of *E. coli*. Representative plate colonies for each treatment group are shown in Figure S1.

### 3.10. Correlation Analysis of Indices Between Dried Shrimp Shell and Meat

**Figure 11:**
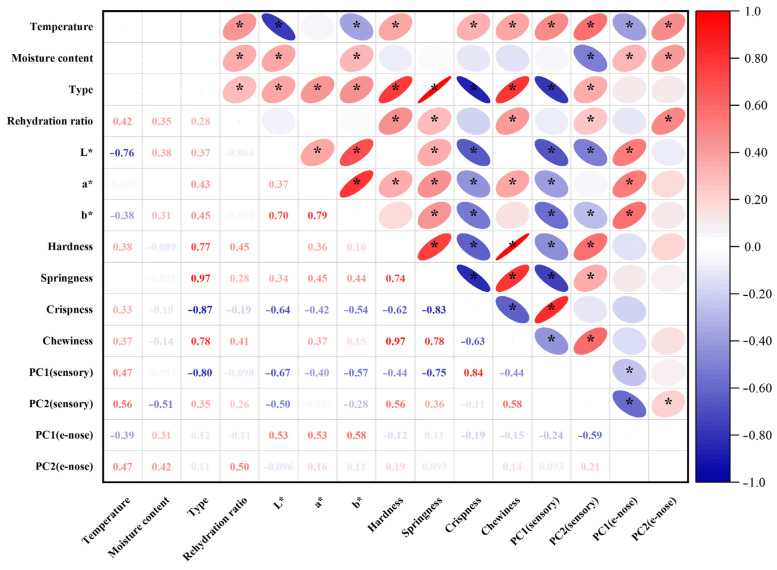
Heatmap of correlation between drying conditions and quality indicators of dried shrimp shell and meat. *: Significance marker for correlation analysis at *p* < 0.05.

The results of the correlation analysis indicated that drying temperature showed significant positive correlations with seven quality indicators: rehydration ratio (*r* = 0.42, *p* < 0.05), hardness (*r* = 0.38, *p* < 0.05), crispness (*r* = 0.33, *p* < 0.05), PC1 (sensory) (*r* = 0.47, *p* < 0.05), PC2 (sensory) (*r* = 0.56, *p* < 0.05), and PC2 (e-nose) (*r* = 0.47, *p* < 0.05) (Figure 11). This indicates that drying temperature, as the primary regulatory parameter, correlates with multiple quality indicators. Specifically, drying temperature showed significant negative correlations with L* (*r* = −0.76, *p* < 0.05), b* (*r* = −0.38, *p* < 0.05), and PC1 (e-nose) (*r* = −0.39, *p* < 0.05). This reflects the impact of high-temperature processing on the Maillard reaction and degradation of heat-sensitive flavor compounds in dried shrimp, resulting in color darkening and flavor deterioration. Moisture content showed significant positive correlations with five indicators: rehydration ratio (*r* = 0.28, *p* < 0.05), L* (*r* = 0.38, *p* < 0.05), b* (*r* = 0.31, *p* < 0.05), PC1 (e-nose) (*r* = 0.31, *p* < 0.05), PC2 (e-nose) (*r* = 0.42, *p* < 0.05). This indicates that dried shrimp tissue with higher moisture content did not undergo excessive shrinkage, retained rehydration capacity, reduced pigment accumulation, and simultaneously minimized degradation of certain heat-sensitive flavor compounds. Conversely, dried shrimp moisture content showed a significant negative correlation with PC2 (sensory) (*r* = −0.51, *p* < 0.05). This suggests that dried shrimp samples with a higher moisture content often exhibit stronger fishy odors. The tissue type showed significant positive correlations with eight indicators: rehydration ratio (*r* = 0.28, *p* < 0.05), L* (*r* = 0.37, *p* < 0.05), a* (*r* = 0.43, *p* < 0.05), b* (*r* = 0.45, *p* < 0.05), hardness (*r* = 0.77, *p* < 0.05), springiness (*r* = 0.97, *p* < 0.05), chewiness (*r* = 0.78, *p* < 0.05), and PC2 (sensory) (*r* = 0.35, *p* < 0.05). It showed significant negative correlations with crispness (*r* = −0.87, *p* < 0.05) and PC1 (sensory) (*r* = −0.80, *p* < 0.05). This discrepancy directly corresponds to the tissue property differences between shrimp shell (thin, brittle tissue) and shrimp meat (high-protein, dense tissue). In summary, tissue type is a key factor determining quality variations during hot-air drying, while drying temperature plays a regulatory role in most quality indicators.

### 3.11. Comparison of Performance Indicators Between Least Squares and LM-ANN Models in Drying Process Prediction

**Figure 12:**
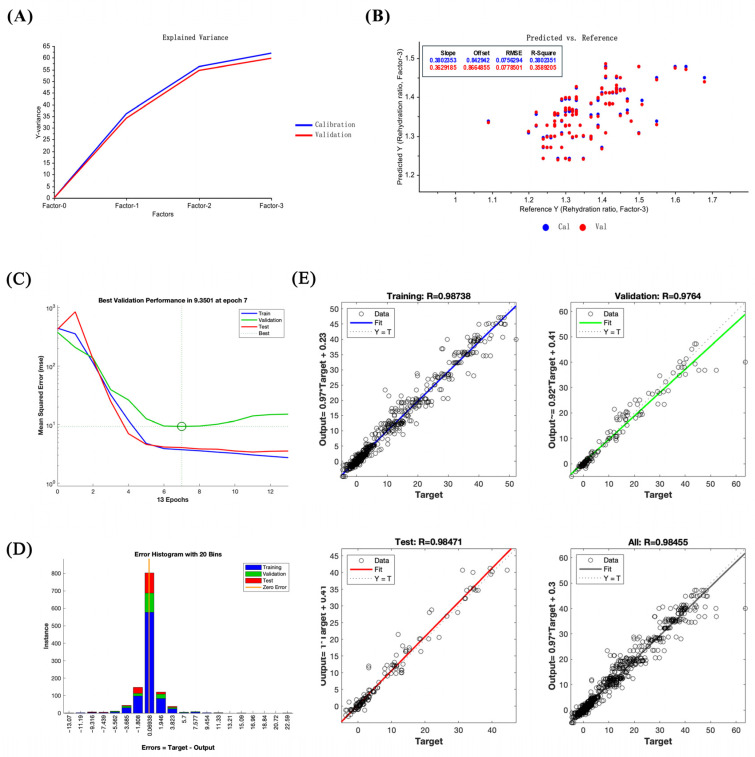
Interaction effects and model establishment for dried shrimp shell and shrimp meat. (**A**) PLSR principal component explaining variance. (**B**) PLSR predicted values versus actual values regression. (**C**) LM-ANN performance curve. (**D**) LM-ANN error histogram, where errors are normalized absolute errors (dimensionless, no physical units) calculated as the difference between target values and predicted values. (**E**) LM-ANN model regression scatter plot, where the *R* is the correlation coefficient between the predicted value and the actual value.

The results of comparing the two models indicate that both models effectively fit the dry shrimp characteristic indicators, though their performance differs. On the calibration dataset, the PLSR linear regression model achieved an R^2^_Calibration_ = 0.38 and RMSE = 0.08. On the validation dataset, R^2^_Validation_ = 0.32 and RMSE = 0.08 (Table 3) (Figure 12A,B). The performance curve of the LM-ANN model indicated that Train, Validation, and Test Lues stabilized near the minimum at epoch 7, confirming model convergence and effective avoidance of overfitting (Figure 12C). The error histogram reveals that most prediction errors cluster between −11.19 and 11.33, exhibiting a quasi-normal distribution (Figure 12D). This indicates overall low prediction errors for the LM-ANN model, confirming its stability in forecasting changes in drying characteristics between shrimp shell and meat. Moreover, in the regression plots of the LM-ANN fitting results, model performance is denoted by the correlation coefficient *R*, whose value reflects the degree of correlation between predicted and actual values. The model achieved correlation coefficients of R_Training_ = 0.99, R_Validation_ = 0.98, R_Test_ = 0.98, and R_All_ = 0.98 (Figure 12E), indicating strong consistency between model predictions and actual observations. Further calculation of the coefficient of determination (R^2^) for the dataset yielded R^2^_Training_ = 0.97 with RMSE = 0.03, R^2^_validation_ = 0.95 with RMSE = 0.04, R^2^_Test_ = 0.96 with RMSE = 0.04, and R^2^_All_ = 0.97 with RMSE = 0.04 (Table 4).

### 3.12. Application of the LM-ANN Model in Predicting Quality Characteristics of Dried Shrimp

Based on the constructed LM-ANN model and considering the characteristics of the data distribution and the practical scenarios of dried shrimp production, this study focused primarily on two core processing components of the same shrimp species (shell and meat) to visually demonstrate the model’s utility. The moisture content of these components was fixed at 15% and 20%, respectively. Different drying temperatures (all input temperatures fell within the 60–100 °C range of the training dataset for this study’s model, constituting interpolation validation based on patterns observed in the training data) were inputted to obtain corresponding dried shrimp quality prediction results (Table 5). The results show that, when the moisture content of the shrimp shell is fixed at 15% or 20% and the input temperatures are 75 °C and 87 °C, and when the moisture content of the shrimp meat is fixed at 15% or 20% and the input temperatures are 64 °C and 93 °C, the quality prediction results for both components align with objective principles and the logic of temperature effects on different tissue qualities. This shows that the LM-ANN model can consistently predict the quality of different tissues of the same shrimp species. It can meet production control requirements for different tissues in dried shrimp factories, thereby having practical application value in such industrial settings.

## 4. Discussion

Changes in quality during the drying process are a significant focus of research within the field of aquatic food processing. Research has shown that the color, texture, and sensory characteristics of shrimp change during the drying process. The drying temperature and method have a significant impact on these changes [33]. Currently, machine learning and neural networks have found extensive application in food drying processes. For example, Al-Hilphy et al. conducted drying experiments on shrimp at different temperatures using a rotary infrared dryer. They employed mathematical models and ANN to predict changes in the wet mass ratio [34]. Niamnuy et al. established an ANN model to predict protein loss and denaturation levels in shrimp during thermal processing, demonstrating that ANNs can effectively capture complex nonlinear relationships between processing conditions and chemical properties such as protein content [35]. These studies provide a viable foundation for adapting similar technologies to different processing factors and realizing quality prediction. The specific processing of different shrimp tissues is crucial for quality, yet most relevant studies neglect the carapace. There is a lack of quality prediction modelling based on actual drying data for both the shrimp shell and meat [36]. Consequently, this study represents the first instance of integrating characteristic data from shrimp shell and meat collected under different hot-air drying conditions into a comprehensive dataset. Using multivariate mathematical modelling techniques such as PLSR and LM-ANN, this study elucidates the differential patterns of quality characteristics across different tissue regions during hot-air drying. This research provides novel methodological and tool-based references for process optimization within the dried shrimp snack food sector. It fills a gap in existing research, particularly regarding dual-organism quality control coordination.

The hot-air drying process significantly affects moisture migration and tissue structure in dried shrimp, highlighting the importance of comparing quality characteristics across different tissues. This study systematically analyses key indicators, including color difference, textural properties, rehydration ratio, sensory evaluation, and electronic nose analysis, to elucidate how hot-air drying affects quality variations among different tissues in dried shrimp. Color is one of the most critical quality indicators of dried seafood products [37]. During the drying process, whole shrimp exhibit a gradient color change from pale grey to red, ultimately transitioning to a deep brown hue. This transformation is indicative of a clear correlation with increasing drying temperatures. The phenomenon under discussion is primarily governed by the combined effects of protein denaturation, astaxanthin release, and the Maillard reaction. On the one hand, the drying heat causes denaturation of the shrimp’s proteins, altering the tissue’s light reflection and absorption properties. Concurrently, denatured proteins release bound astaxanthin, thereby imparting a vivid red hue to the entire shrimp. As the drying process continues, Maillard reactions between reducing sugars and amino acids within the shrimp lead to the production of brown melanin-like compounds, resulting in a progressive deepening of the color to dark brown. This mechanism also provides a plausible explanation for the characteristic black tint observed in separated shrimp shell. The red-orange pigmentation of shrimp is primarily attributed to the deposition of xanthophylls, including β-carotene, astaxanthin, canthaxanthin, and related carotenoid compounds [38]. The a* and b* values of dried shrimp meat increased during hot-air drying. It is speculated that this may be related to increased release of carotenoids, such as astaxanthin, due to thermal effects during the drying process. Niamnuy et al. observed that the increase in a* and b* values in color difference parameters during drying and storage was closely correlated with astaxanthin content in shrimp [39]. However, this association has not yet been directly verified in the present study and warrants further investigation. The textural properties of dried aquatic products, such as hardness, springiness, chewiness, and crispness, are key indicators of quality and palatability. Both shell and meat hardness increased with higher drying temperatures, likely due to protein denaturation and tissue contraction causing structural densification. The higher meat hardness compared to shell hardness may be attributed to the gradual moisture loss during hot-air drying, causing dissociation of actin and myosin, which enhances the rigidity of muscle fiber architecture and ultimately induces surface hardening [21]. The brittleness of shrimp shell increases with rising drying temperatures, peaking at 4.61 N. This increase in brittleness is primarily due to the presence of chitin in shrimp shell. Higher drying temperatures reduce the interaction between the molecular chains of chitin, resulting in decreased tensile strength and a more brittle shell structure [40]. Additionally, the chewiness of both shrimp shell and meat progressively increases during drying, reaching maximum values of 3.00 N and 28.94 N, respectively. This occurs because proteins denature at high temperatures, while the increased temperature reduces tissue porosity, thereby enhancing chewing resistance. Xiong et al. also confirmed that hot-air drying simultaneously enhances both hardness and chewiness [41]. The springiness of shrimp meat ranges from 0.72 to 0.84 newtons. This indicates that the protein structure within the meat may remain relatively stable within the selected temperature range. Other comparable research has also verified this point [21]. Overall, shrimp meat outperformed shrimp shell in multiple textural parameter results, indicating a more compact structure. Rehydration is the process by which dried products absorb moisture again and return to their original state. This serves as a key indicator of the quality of dehydrated foods. As drying temperature increased and moisture content decreased, the rehydration ratio of the shrimp shell showed no significant variation between groups. Conversely, the rehydration ratio of the shrimp meat gradually increases, reaching a maximum of 1.51. This discrepancy stems from the fact that shrimp meat is more prone to cracking and porosity during hot-air drying, which results in superior rehydration performance. In contrast, the chitin in shrimp shell becomes denser when dried, reducing water vapor permeability and restricting inward moisture diffusion, which results in a lower overall rehydration rate [40]. However, when dried to lower moisture contents, protein aggregates contract more, which causes the internal pores of the dried shrimp bodies to close more completely and consequently reduces their rehydration capacity. Niamnuy et al. have indicated that a higher degree of protein contraction may reduce the rehydration capacity of mechanically dried shrimp meat [33]. Sensory evaluation is a reliable quantitative method for quantifying the odor profiles of food. Sensory analysis results indicate that shrimp shell exhibit a more pronounced roasted/nutty aroma during hot-air drying, with a peak value of 4.60/5.00. Zhang et al.’s research also shows that shrimp exoskeletons contain a lot of substances that can be used to make aromatic compounds. These substances play a big part in making volatile compounds [31]. During hot-air drying processing, the shrimp meat retained some fishy flavor, peaking at 3.39/5.00. This may stem from the concurrent occurrence of the Maillard reaction, lipid oxidation, and nitrogen-source decomposition during hot-air drying. Previous studies indicate that lipid oxidation in sun-dried fish produces both nutty-flavored aldehydes and fishy odors characterized by heptanal, 2-octenal, and 2-heptenal (E) [42]. The electronic nose employs an array of gas sensors to objectively characterize changes in volatile flavor compounds within shrimp products by simulating the human olfactory system’s recognition of complex odors. This allows differences in flavor formation across distinct dried shrimp tissues to be analyzed. Electronic nose results indicate that shrimp meat with lower moisture content exhibits stronger response values than shrimp shell on the W1W metal oxide gas sensor. This is due to lipid oxidation and Maillard reactions occurring during drying, which generate an abundance of aromatic volatiles and certain aldehydes. The concentration of these compounds significantly exceeds that in shrimp shell, which are primarily composed of chitin, thereby elevating the W1W component’s response to shrimp meat. Zheng et al. observed that drying shrimp yields multiple pyrazine-type aromatic compounds, the concentrations of which increased markedly during processing. Pyrazines are typically associated with nutty and roasted aromas [43]. It is noteworthy that the W1W sensor exhibits a pronounced response at approximately 70 °C, which subsequently diminishes as the temperature continues to rise. This trend may be associated with phased alterations in the composition of volatile constituents. The low-molecular-weight sulfur-containing compounds and certain short-chain aldehydes generated during the initial heating phase constitute the primary targets of W1W sensitivity [44]. At higher temperature, these compounds may diminish due to further involvement in Maillard reactions or thermal degradation. The subsequent formation of macromolecular heterocyclic products elicits a weaker effective response from W1W, thereby reducing the signal. As the e-nose provides only a composite response and cannot directly distinguish specific volatiles, this phenomenon requires further confirmation through GC–MS compositional analysis. The Pearson correlation analysis was performed to clarify the relationship between drying conditions and quality indicators in dried shrimp. This revealed strong positive correlations between tissue type, elasticity, and hardness, as well as significant negative correlations with brittleness. These results directly reflect the differences in tissue between shrimp shell and meat. Drying temperature showed significant positive correlations with most indicators, confirming the impact of high temperatures on color and flavor. Moisture content showed positive correlations with rehydration rate and negative correlations with fishy odor. In summary, tissue type is the key determinant of quality variation, while drying temperature is the primary regulatory parameter.

Mathematical models were introduced to further reveal the multivariate relationships among various tissues in dried shrimp, based on the quality changes of different tissues in shrimp shell and meat during hot-air drying. As a typical linear regression model, PLSR effectively addresses issues of multicollinearity among variables. This yields an R^2^_Calibration_ of 0.38 and an RMSE of 0.08 for the calibration set. The validation set yielded an R^2^_Validation_ of 0.32 and an RMSE of 0.08. While this model can partially identify linear relationships between temperature, moisture content and quality parameters, it is limited in its ability to predict non-linear changes. The neural network optimized using the Levenberg–Marquardt algorithm demonstrated exceptional accuracy in handling non-linear fitting. The model’s prediction correlation coefficients with actual values were R_Training_ *=* 0.99 and R_Validation_ = 0.98. The training and validation set yielded R^2^ and RMSE of R^2^_Training_ = 0.97, RMSE = 0.03 and R^2^_Validation_ = 0.95, RMSE = 0.04. This study validated the superiority of LM-ANN over PLSR for fitting data. The practical application of the LM-ANN model’s performance advantages depends on implementing interactive feedback loops. The MATLAB Neural Network Toolbox, with custom core code added, is the tool of choice for this study. It processes inputs such as tissue type, drying temperature, and moisture content and predicts 12 indicators of dried shrimp quality. This allows for direct integration with production engineering scenarios for adjusting process parameters. This kind of tool-based functionality is more likely to be adopted widely, providing technical guidance for intelligent and precise control in dried shrimp processing. However, the LM-ANN model currently in use has certain limitations. For example, it struggles to accurately predict subtle variations in specific aromatic compounds, such as aldehydes and sulfur-containing compounds, within dried shrimp during processing. Similarly, it has difficulty capturing deeper indicators related to texture, such as proteolytic changes and alterations in fiber structure. Its ability to capture and forecast such nuances is limited. Furthermore, evaluations of the characteristic aroma and core texture of dried shrimp based solely on existing data yield rather generalized results. Integrating physicochemical testing data from spectroscopic analysis techniques such as GC-MS and HPLC could significantly improve the accuracy of predicting these characteristics. Subsequent work will continue this integrated technical approach, combining high-temperature-resistant fiber optic probes with the oven environment to construct a multi-channel sensor array utilizing near-infrared spectroscopy technology. This will enable the real-time, non-contact monitoring of quality indicators during dried shrimp processing, including moisture content, color changes, and protein denaturation. This addresses the inherent delays in conventional detection methods. Concurrently, data from other species such as *Pandalus platyceros* (Spot prawns) and *Macrobrachium rosenbergii* (Giant river prawns) will be incorporated to optimize the model’s adaptability across diverse shrimp processing scenarios.

## 5. Conclusions

This study systematically compared the quality differences between the shell and meat of various tissues of dried shrimp under hot-air drying conditions. An in-depth analysis was conducted of the drying characteristics and quality change patterns. Furthermore, to address the need for precise prediction under these conditions, PLSR and LM-ANN models were constructed and their performance was compared. It was determined that within the hot-air drying parameters of this study, LM-ANN serves as a tool with high predictive performance in dried shrimp production. This tool can input tissue type, drying temperature, and moisture content to generate predicted values for target quality indicators. The results indicate that shrimp shell and meat exhibit differences in drying rate, textural properties, rehydration performance, color changes, and sensory characteristics under different drying conditions. This shows that the hot-air drying process affects different tissue structures within dried shrimp in different ways. Specifically, the firmness of the shrimp meat reaches its peak at 100 °C, making it ideal for production requirements seeking a firm, chewy texture. The roasted/nutty flavor profile of the shrimp shell is most pronounced at 100 °C, better catering to flavor-driven production scenarios. Based on these findings, the LM-ANN model demonstrated significantly superior predictive performance for different tissue qualities compared to the PLSR model. The training set R^2^_Training_ was 0.97 and the validation set R^2^_Validation_ was 0.95, with corresponding RMSEs of 0.03 and 0.04, respectively. In contrast, the PLSR model achieved a calibration set R^2^_Calibration_ of 0.38 and a validation set R^2^_Validation_ of 0.32, with corresponding RMSEs of 0.08 for both. These results demonstrate that the LM-ANN model is more effective at fitting nonlinear data and complex interactions, while also offering greater interpretability. This model is a reliable tool for analyzing quality variations in dried shrimp shell and meat. Subsequent research may use near-infrared spectroscopy to enable the real-time monitoring of important quality indicators in dried shrimp. Integrating HPLC astaxanthin determination and GC-MS volatile compound analysis will supplement the model with critical quality data, optimize input parameters, and increase its applicability to different species of shrimp and drying processes. This approach shows promise in terms of achieving real-time quality prediction and precise process control in dried shrimp production.

## Figures and Tables

**Table 1:** Summary of distribution of experimental samples for dried shrimp components.

Sample Type	Total Sample Size	Non-Destructive (Quantity)	Destructive (Quantity)
Shrimp shell	25	Visual inspection and color difference analysis (5)	Rehydration ratio, sensory evaluation, e-nose analysis, TPA (20, each 5)
Shrimp meat	25	Visual inspection and color difference analysis (5)	Rehydration ratio, sensory evaluation, e-nose analysis, TPA (20, each 5)

**Table 2:** Main parameter settings of the LM-ANN model.

Parameter	Setting
Network type	Feed-forward backpropagation neural network
Neurons in hidden layers	10
Input variables	3
Output variables	12
Activation function (hidden layer)	Sigmoid (logsig)
Activation function (output layer)	Linear (purelin)
Epochs (maximum iterations)	1000
Training algorithm	Levenberg–Marquardt
Data division	70% training, 15% validation, 15% testing
Random seed	1

**Table 3:** Performance evaluation metrics of the PLSR model on different data subsets.

Dataset Type	R^2^	RMSE
Calibration	0.38	0.08
Validation	0.32	0.08

**Table 4:** Performance evaluation metrics of the LM-ANN model on different data subsets.

Dataset Type	R	R^2^	RMSE
Training	0.99	0.97	0.03
Validation	0.98	0.95	0.04
Test	0.98	0.96	0.04
All	0.98	0.97	0.04

Note: *R* denotes the correlation coefficient measuring the linear relationship between the LM-ANN predicted values and the actual values, while R^2^ denotes the coefficient of determination measuring the model’s goodness of fit.

**Table 5:** Example data for predicting dried shrimp quality based on the LM-ANN model.

Input Parameters	Prediction Case A	Prediction Case B	Prediction Case C	Prediction Case D
Tissue type	0	0	1	1
Temperature (°C)	75	87	64	93
Moisture content (%)	20	15	20	15
Predictive indicators	Prediction Case A	Prediction Case B	Prediction Case C	Prediction Case D
Rehydration ratio	1.34	1.26	1.38	1.34
L* (lightness)	37.44	28.71	42.91	31.04
a* (redness)	13.13	11.60	11.19	11.41
b* (yellowness)	15.57	12.36	18.32	11.35
Hardness	4.33	5.24	11.40	39.32
Springiness	0.33	0.48	0.63	0.79
Crispness	2.50	3.49	0.51	0.46
Chewiness	0.98	2.94	6.69	28.58
PC1 (sensory)	0.74	1.89	−0.99	−0.76
PC2 (sensory)	−1.58	0.90	−1.23	2.09
PC1 (e-nose)	2.39	−2.34	0.86	−4.08
PC2 (e-nose)	0.08	−0.25	−0.39	−0.45

## Data Availability

The original contributions presented in this study are included in the article/Supplementary Materials. Further inquiries can be directed to the corresponding authors.

## Supplementary Materials

The following supporting information can be downloaded at: https://www.mdpi.com/article/10.3390/foods14234041/s1, Table S1: Definitions of sensory attributes for hot-air dried shrimp samples; Table S2: E-nose sensors and their main application in PEN3; Table S3: PLSR regression coefficients for physicochemical quality attributes; Table S4: PLSR regression coefficients for odor attributes; Document S1: The code for LM-ANN to implement variable input and prediction output; Figure S1: Microbiological indicator test results for dried shrimp shell and shrimp meat (10^−2^ dilution).
