# Supplementary material for: Effects of Hot-Air Drying Conditions on Quality Attributes of Meat and Shell of Dried Shrimp
Source: Foods. 2025 Nov 25;14(23):4041. doi: 10.3390/foods14234041 (PMC12691823; doi:10.3390/foods14234041)
Supplement: Supplementary file 1 [file foods-14-04041-s001.zip › foods-3969409-supplementary.pdf]

Table S1. Definitions of sensory attributes for hot-air dried shrimp samples.

Sensory attribute terminology	Qualitative definition
Metallic ((Z)-1,5-octadien-3-one)	Metal objects, unpleasant odor
Sweet (maltol)	Fruity, sweet note. Taste and smell of low-carbon ester
Cooked-meat-like (3-(methylthio) propanal)	Roasted meat, baked potato, fried chicken
Fishy (trimethylamine)	Fresh seaweed or marine
Caramel (4-hydroxy-2,5-dimethyl-3(2H)-furanone)	Pleasant honey and caramel odors
Roasted/nutty (2,5-dimethylpyrazine)	Smells like toasted peanuts, hazelnuts, popcorn, almonds and chocolate.

Table S2. E-nose sensors and their main application in PEN3.

Serial No.	Sensor Name	Representative Material Species
1	W1C	Aromatic compounds
2	W5S	Broad range
3	W3C	Aromatic
4	W6S	Hydrogen
5	W5C	Arom-aliph
6	W1S	Broad-methane
7	W1W	Sulphur-organic
8	W2S	Broad-alcohol
9	W2W	Sulph-chlor
10	W3S	Methane-aliph

Table S3. PLSR regression coefficients for physicochemical quality attributes.

Predictor variable	Rehydration ratio	L*	a*	b*	Hardness	Springiness	Crispness	Chewiness
b_0	0.87	44.31	11.58	12.58	-20.77	0.36	1.31	-10.48
Temperature	0.00	-0.36	-0.02	-0.13	0.44	0.00	0.03	0.29
Moisture content	0.01	1.01	0.05	0.611	-0.59	0.00	-0.06	-0.63
Tissue type	0.05	4.93	3.49	4.42	25.62	0.45	-2.44	17.51

Note: b_0 denotes the regression coefficient.

Table S4. PLSR regression coefficients for odor attributes.

Predictor variable	PC1 (sensory)	PC2 (sensory)	PC1 (e-nose)	PC2 (e-nose)
b_0	-2.40	0.03	-0.12	-5.93
Temperature	0.06	0.05	-0.08	0.03
Moisture content	-0.04	-0.25	0.34	0.17
Tissue type	-2.66	0.84	0.64	0.23

Supplementary Document S1. The code for LM-ANN to implement variable input and prediction output.

The supplementary code details are as follows, assuming the input values are $x_1 = 1$, $x_2 = 64$, and $x_3 = 20$.

```
% Input Parameter Definition

%   x_new(1)：Tissue type (0=Shrimp shell, 1=Shrimp meat)

%   x_new(2)：Temperature (°C)

%   x_new(3)：Moisture content (%)

x_new = [1;      % Tissue Type = Meat

      64;     % Temperature = 64°C

      20];     % Moisture content = 20%

% Model Prediction

% Input: 3×1 feature vector x_new

% Output: 12×1 vector y_new

y_new = net(x_new);

% Prediction results output

fprintf('1. Rehydration ratio= %.2f\n', y_new(1));

fprintf('2. L* = %.2f\n', y_new(2));

fprintf('3. a* = %.2f\n', y_new(3));

fprintf('4. b* = %.2f\n', y_new(4));

fprintf('5. Hardness = %.2f\n', y_new(5));

fprintf('6. Springiness = %.2f\n', y_new(6));

fprintf('7. Crispness = %.2f\n', y_new(7));

fprintf('8. Chewiness = %.2f\n', y_new(8));

fprintf('9. PC1(sensory) = %.2f\n', y_new(9));

fprintf('10. PC2(sensory) = %.2f\n', y_new(10));

fprintf('11. PC1(e-nose) = %.2f\n', y_new(11));

fprintf('12. PC2(e-nose) = %.2f\n', y_new(12));
```

Note: In MATLAB, % is the comment symbol. x_new denotes the newly input variable. y_new denotes the newly predicted output result. fprintf is a function used for formatted output, displaying data on the screen according to a specified format. %.2f indicates output with two decimal places retained. \n signifies a newline, used to make printed results more organized and readable.

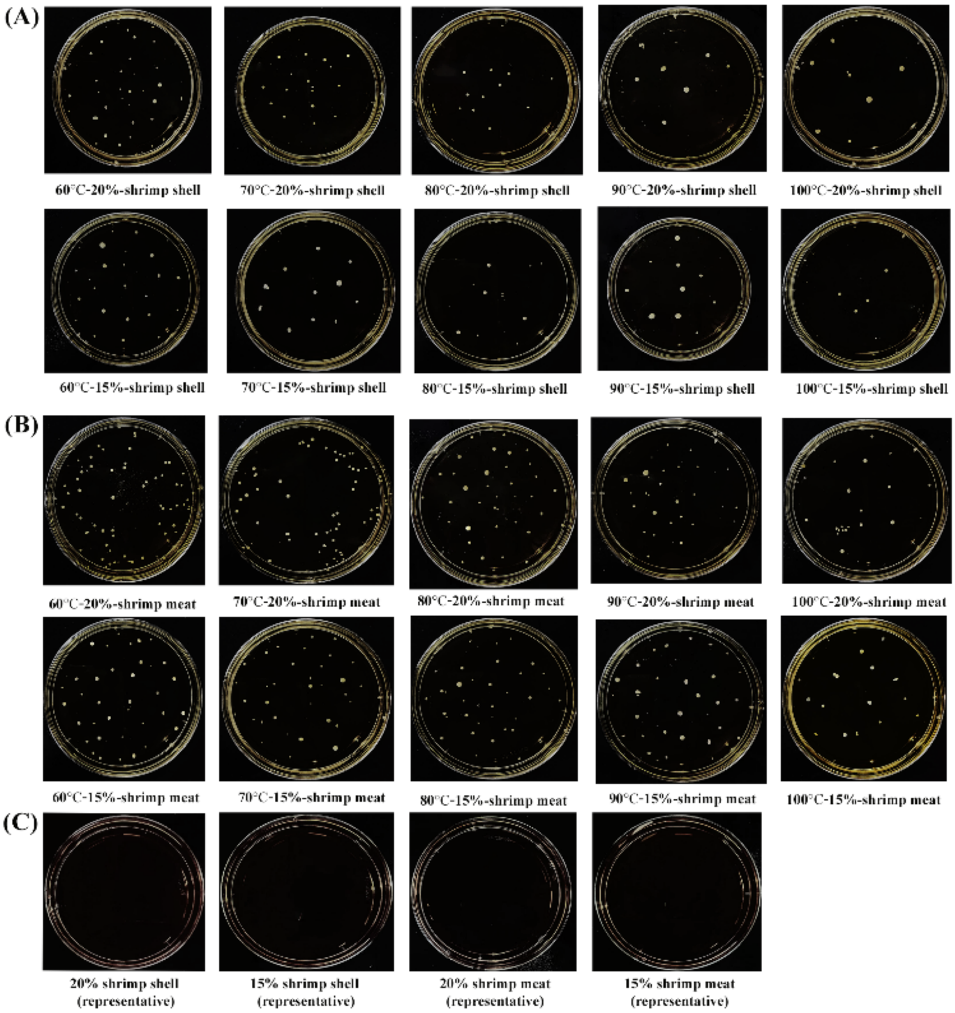

Figure S1. Microbiological indicator test results for dried shrimp shell and shrimp meat (10^{-2} dilution). (A) Total colony count detection results for shrimp shell using PCA; (B) Total colony count detection results for shrimp meat using PCA; (C) Representative blank plates from VRBA agar plates (corresponding to VRBA testing of shrimp shell and shrimp meat, with no typical coliform colonies observed in either).
